# Accurate Determination of the Frequency Response Function of Submerged and Confined Structures by Using PZT-Patches †

**DOI:** 10.3390/s17030660

**Published:** 2017-03-22

**Authors:** Alexandre Presas, David Valentin, Eduard Egusquiza, Carme Valero, Mònica Egusquiza, Matias Bossio

**Affiliations:** CDIF (Centre de Diagnòstic Industrial I Fluidodinàmica), UPC (Universitat Politècnica de Catalunya), Av. Diagonal 647, 08028 Barcelona, Spain; david.valentin@upc.edu (D.V.); eduard.egusquiza@upc.edu (E.E.); valero@mf.upc.edu (C.V.); monica.egusquiza@upc.edu (M.E.); matiasbossio1@gmail.com (M.B.)

**Keywords:** PZT actuators, modal analysis, exciters, submerged structures

## Abstract

To accurately determine the dynamic response of a structure is of relevant interest in many engineering applications. Particularly, it is of paramount importance to determine the Frequency Response Function (FRF) for structures subjected to dynamic loads in order to avoid resonance and fatigue problems that can drastically reduce their useful life. One challenging case is the experimental determination of the FRF of submerged and confined structures, such as hydraulic turbines, which are greatly affected by dynamic problems as reported in many cases in the past. The utilization of classical and calibrated exciters such as instrumented hammers or shakers to determine the FRF in such structures can be very complex due to the confinement of the structure and because their use can disturb the boundary conditions affecting the experimental results. For such cases, Piezoelectric Patches (PZTs), which are very light, thin and small, could be a very good option. Nevertheless, the main drawback of these exciters is that the calibration as dynamic force transducers (relationship voltage/force) has not been successfully obtained in the past. Therefore, in this paper, a method to accurately determine the FRF of submerged and confined structures by using PZTs is developed and validated. The method consists of experimentally determining some characteristic parameters that define the FRF, with an uncalibrated PZT exciting the structure. These parameters, which have been experimentally determined, are then introduced in a validated numerical model of the tested structure. In this way, the FRF of the structure can be estimated with good accuracy. With respect to previous studies, where only the natural frequencies and mode shapes were considered, this paper discuss and experimentally proves the best excitation characteristic to obtain also the damping ratios and proposes a procedure to fully determine the FRF. The method proposed here has been validated for the structure vibrating in air comparing the FRF experimentally obtained with a calibrated exciter (impact Hammer) and the FRF obtained with the described method. Finally, the same methodology has been applied for the structure submerged and close to a rigid wall, where it is extremely important to not modify the boundary conditions for an accurate determination of the FRF. As experimentally shown in this paper, in such cases, the use of PZTs combined with the proposed methodology gives much more accurate estimations of the FRF than other calibrated exciters typically used for the same purpose. Therefore, the validated methodology proposed in this paper can be used to obtain the FRF of a generic submerged and confined structure, without a previous calibration of the PZT.

## 1. Introduction

In many engineering applications, it is of paramount importance to accurately determine the dynamic structural response of a structure (or assembly) in order to avoid dynamic problems such as resonances or fatigue that can drastically reduce their useful life. Modal analysis is a technique that has been widely used in the past for this purpose [1,2,3,4]. One of the main outputs of the modal analysis is the Frequency Response Function (FRF), which is the relationship between the response of the structure (in terms of vibration) and the dynamic force. This is particularly important when analyzing the dynamic response of the structure due to an exciting force with a frequency content close to one of the natural frequencies of the structure, since the response of the structure can be determined with precision and therefore resonance or fatigue problems, which have caused several failures in the past [5,6], could be avoided.

When performing modal analysis, one of the crucial points is the selection of the actuators that will excite the tested structure. Typical requirements for these exciters are a good performance signal/noise ratio for the frequency range of interest [2] and that they do not affect the estimated modal parameters (and therefore the FRF) of the tested structure [7]. Furthermore, the sensitivity of the transducer, i.e., relationship between electrical and mechanical signal, has to be known. Typical actuators that are used for modal analysis are instrumented hammers, which are not fixed to the structure and many different types of shakers (fixed or not to the structure). For these exciters, the sensitivity is generally known (given by the manufacturer) and therefore the force that is being applied to the structure can be directly obtained from the electrical signal. Although these exciters have been proven to be good options for modal analysis in simple and accessible structures, for inaccessible structures (such as rotating, confined and/or submerged structures), it can be very difficult to use these classical exciters due to the inaccessibility of the structure. Therefore, Piezoelectric Patches (PZTs), which are extremely light and thin, could be used to determine the FRF.

In the past, PZTs have been used as exciters proving that natural frequencies and mode shapes can be properly determined. Particularly, plenty of studies can be found where PZTs have been used for structures vibrating in air [8,9,10,11,12,13,14]. Wang [8] and Yan [9] used PZTs for active control vibration in order to reduce the flutter in a thin disk. Yang [10] and El Sekouri [11] determined in their respective studies the natural frequencies and mode shapes of thin plates vibrating in by using PZTs. Cheng [12] used several patches to excite a thin structure and estimated the added mass effect of those ones. These studies are mainly concerned with very thin disks such as storage disks. More recently, Presas [13] used several patches to excite a relative stiff and rotating disk with several excitation patterns that really occur in rotating turbomachinery. Nevertheless, in that study, only the natural frequencies and mode shapes of the structure are evaluated, but the damping ratios and scaling factors of the vibration modes were not determined. Finally, Gomis-Bellmunt [14] mentioned the main advantages and drawbacks of using PZTs as exciters and particularly the hysteresis of PZTs is evaluated in that study. Although during the last decade extensive work has been performed using PZTs as exciters for active/passive vibration of several kinds of structures, accurate determination of the FRF of those structures with these exciters has not been discussed yet.

PZTs are particularly interesting to determine structural parameters (such as natural frequencies and mode shapes) of submerged structures or for the active vibration control of such structures, since other kind of exciters are difficult to be used due to inaccessibility of the structure. Therefore, PZTs have been successfully used in the last years [13,15,16,17,18,19,20]. Presas et al. [13,15,16] successfully determined the natural frequencies and mode shapes of a rotating and confined stiff disk submerged and confined in water by means of PZT. In [17], the natural frequencies and mode shapes of a reduced scale model of a real hydraulic turbine could be determined by using PZTs. Furthermore, De la Torre [18] used a PZT to determine the same structural parameters of a submerged hydrofoil under cavitating conditions. In all of those cases, it was not possible to use classical exciters due to the inaccessibility of the respective structures. Recently Kwak [19,20] successfully used PZTs as actuators and sensors for an active vibration control of a partially submerged thin plate.

The review of the extensive work that has been performed during the last decade with PZTs actuators leads to the main conclusion that PZTs have been successfully used for the active vibration control of thin structures vibrating in air and in water and for the determination of natural frequencies and mode shapes of submerged structures. Nevertheless, the use of these exciters to determine the FRF (which implies to determine also damping ratios and scaling factors [2]), of a generic structure has not been discussed yet. Possible reasons are the non-linear behavior of these actuators [14] which means that the sensitivity of the actuator in terms of dynamic force is not generally known for such exciters. Furthermore, for a generic structure without confinement, it is also possible and easier to experimentally use other calibrated exciters to determine the FRF [21]. Therefore, for such structures (vibrating in air and not confined), it is maybe unnecessary to use PZTs due to its drawbacks compared to other linear actuators, such as instrumented hammers. Nevertheless, to determine the FRF of a generic stiff structure submerged in water and confined, the use of exciters such as PZTs (light, thin and small) can become almost fundamental, since the aforementioned classical exciters are much more difficult to be physically used (due to its inaccessibility) and the use of them may modify the real FRF of the structure.

Therefore, the objective of this paper is to evaluate the use of PZT-patches to determine the FRF of a submerged structure with nearby rigid walls and to show that the use of these exciters is very advantageous in front of other classical exciters. The accurate determination of the FRF implies also to accurately determine the damping factors of the structure in those conditions, which have not been determined in previous studies. In Section 2, the generic modal analysis model is presented. In Section 3, the equipment used and the experimentation done with the tested structure is explained. Section 4 experimentally shows the proposed method in this study to obtain the FRF of a generic structure by using PZTs. In Section 5, which shows the interest and potentiality of this research, the methodology presented in the previous section is applied for the same structure submerged with nearby rigid walls. Finally, Section 6 is the conclusions and future perspectives of the present work.

## 2. Modal Analysis Model

### 2.1. Generic Equations for Modal Analysis

This section briefly presents the model inherent in the modal analysis. The objective of this is simply to see which parameters take place in the Frequency Response Function (FRF) or relation between displacement and exciting force.

According to the modal analysis model, each structure can be understood as a superposition of masses, springs and dampers interconnected to each other. The matrix form of the structural equation subjected to a generic dynamic load can be expressed as [1,2]:

(1) $$\left[M\right]\ddot{x}\left(t\right)+\left[C\right]\dot{x}\left(t\right)+\left[K\right]x\left(t\right)=F\left(t\right),\;\mathrm{where}\;x=\left\{\begin{array}{c} x_{1}\\ x_{2}\\ \vdots \\ x_{n} \end{array}\right\}$$

$x\left(t\right),\;\dot{x}\left(t\right)$, and $\ddot{x}\left(t\right)$ are, respectively, the displacement, velocity and acceleration in the *n* discretized points or degrees of freedom (DOF) in the time domain. [M], [C], [K] are, respectively, the matrices of mass, dampers and springs. They represent the interconnection existing in the analyzed structure of the different points or DOFs. F(t) is a vector (with the same size than *x*) that express the force applied on each DOF in the time domain.

The Frequency response Function (FRF) can be calculated by transforming Equation (1) in the frequency domain (*jω*) by means of the Fourier Transform:

(2) {X(jω)}=[H(jω)]·{F(jω)}

{X(jω)}, {F(jω)} are the corresponding vectors x(t) and F(t) in the frequency domain. Thus, the FRF, or relationship between {X(jω)} and {F(jω)}, is the matrix [H(jω)]. This matrix has dimension *n* × *n*. An element of this matrix, for instance *h_a,b_*, can be understood as the relationship existing between the displacement of the DOF *a (x_a_*) when a force is applied on the DOF *b (F_b_)*.

The FRF of a proportionally damped system (which is a validated model of damping in many real structures) can be also expressed as [1,2]:

(3) $$\left[H\left(j\omega \right)\right]=\overset{N}{\underset{r=1}{\sum }}\frac{j2\omega_{r}Q_{r}{\left\{\vartheta \right\}}_{r}{{\left\{\vartheta \right\}}_{r}}^{t}}{\left(\theta_{r}^{2}+{\omega_{r}}^{2}-{\omega_{\;}}^{2}\right)-2\theta_{r}j\omega }$$

N represents the number of vibration modes (in fact N=n). Equation (3) is expressed as the superposition of these vibration modes. Each vibration mode *r* is defined by the following modal parameters:

- Natural frequency: $\omega_{r}$ is the natural frequency of the corresponding mode *r*.
- Damping: $\theta_{r}$ is the damping factor and determines the amplitude of the structural response when the system is close to a resonance condition $\omega =\omega_{r}$.
- Mode shapes: ${\left\{\vartheta \right\}}_{r}$ define the deformation shape that dominates the structure close to resonance condition $\omega =\omega_{r}$.
- Scaling Factor: $Q_{r}$ is a constant factor defined for each mode *r* and has an influence on the amplitude of the response.

The FRF can be also represented as the relation between velocity of vibration and force ($\left\{\dot{X}\left(j\omega \right)\right\},\;\left\{F\left(j\omega \right)\right\}$) or Mobility Function [H(jω)]′ and also as the relation between acceleration and force ($\left\{\ddot{X}\left(j\omega \right)\right\},\;\left\{F\left(j\omega \right)\right\}$ or Inertance [H(jω)]″ [1]. They are easily related to each other as shown in Equation (4) (deduced also in [1]) and they are equally valid forms of the FRF.

In the present work, the word FRF will be used for the Inertance, since experimental measurements will be made by means of an accelerometer (Section 3). Without loss of validity, the conclusions and comments made in this paper for the vector {X(jω)} are equivalent to those for vector $\left\{\ddot{X}\left(j\omega \right)\right\}$ and therefore, for clarity of the explanations, only vector {X(jω)} will be used.

(4) $$\left[H\left(j\omega \right)\right]=\frac{{\left[H\left(j\omega \right)\right]}^{\prime }}{j\omega }=\frac{{\left[H\left(j\omega \right)\right]}^{\prime \prime }}{-\omega^{2}}$$

Usually, in Experimental Modal Analysis (EMA), the vectors {X(jω)}, {F(jω)} are measured simultaneously with appropriate transducers. With these two vectors, [H(jω)] can be determined and therefore the associated modal parameters.

Generally, {X(jω)} is obtained with a motion sensor as an accelerometer (fixed to the structure) or with a non-contact sensor (proximity probe, Laser Doppler Vibrometer, etc.). To measure {F(jω)}, a calibrated transducer, i.e., sensitivity of the sensor in force *(V*/*N)*, is needed. Typically used exciters, due to their linearity in the sensitivity value, are instrumented hammers.

When using another kind of exciters, such as PZT-patches, this sensitivity is not linear and not given by the manufacturers. According to the authors knowledge, this sensitivity has not been successfully obtained in the past.

### 2.2. Comments for Fluid-Structure Interaction Problems

The general equations presented above are valid for a generic and arbitrary structure. Nevertheless, the matrixes [M],[C],[K] can include complex and not linear terms (see for example [15]) that may come from the boundary conditions of the structure, such as the surrounding fluid.

Without losing generality, the case of a fluid–structure interaction problem can be described as follows (Equation (5)):

(5) $$\left[M+M_{F}\right]\ddot{x}\left(t\right)+\left[C+C_{F}\right]\dot{x}\left(t\right)+\left[K+K_{F}\right]x\left(t\right)=F\left(t\right),$$

In this case, the solution of Equation (5) leads to the natural frequencies, mode shapes and damping ratios of the coupled fluid–structure system. During the last years, this problem has been extensively investigated for structures vibrating in a heavy fluid [15,22,23,24,25,26,27], such as water. It is out of the scope of this paper to analyze the fluid–structure interaction problem, but it is important to mention the main changes in the structural response of a generic structure when it is totally submerged in a heavy fluid.

Generally speaking, it is founded that when the structure is submerged in a heavy fluid the added mass effect ($M_{F}$) reduces the natural frequencies. This effect is even more important (larger $M_{F}$) when the structure is approaching to a rigid wall [28,29,30,31]. Recent results show that the fluid damping $C_{F}$ also depends on the distance to the rigid wall [28,32]. It can be generally stated that for a structure totally submerged in a heavy fluid, when the distance to a rigid wall decrease, the natural frequencies generally decrease and the damping ratios increase.

From the aforementioned references, it can be concluded that when the submerged structure is close to one or more rigid walls, a small change in the gap structure–wall (or its derivatives) could drastically affect the correct estimation of the modal parameters. From the experimental point of view, this implies that if the FRF of a submerged structure close to rigid walls has to be determined with precision, the distance to the nearby rigid wall has to be maintained as constant as possible in order to not affect $M_{F}$ and $C_{F}$. Nevertheless, this can be very complicated if a classical exciter is used, since an impact on the tested structure may moves it towards the wall, affecting the boundary conditions.

### 2.3. Generic Equations for a PZT-Actuator

The constitutive equations for a generic piezoelectric transducer are well known and have been presented in many studies (see for instance [33,34,35]). For a discrete piezoelectric transducer formed by a stack of *n* sheets, with electrical and mechanical properties uniformed distributed, these equations can be presented in a generic form as [34]:

(6) $$\left\{\begin{array}{c} Q\\ \Delta \end{array}\right\}=\left[\begin{array}{cc} C & nd_{33}\\ nd_{33} & 1/K_{a} \end{array}\right]\left\{\begin{array}{c} V\\ F \end{array}\right\}$$

where *Q* is the total electric charge on the electrodes of the transducer and Δ the total extension of the transducer. V is the voltage applied between electrodes and F the total force. *C* is the capacitance of the transducer with no external loads and $K_{a}$ the stiffness of the transducer for *V* = 0. $d_{33}$ is a piezoelectric constant that is used in this equation assuming that electric field is parallel to the poling direction of the transducer.

When the PZT-actuator is attached (glued) on a “tested structure” and a voltage is applied between electrodes, the static force and the total extension of the transducer depend on the material and thickness of the “tested structure”. For the PZT-actuator, the dynamic force also depends on the working frequency [33].

Therefore, in front of classical exciters used in modal analysis such as instrumented hammers where the relationship between voltage and dynamic force is linear and independent from the tested structure, for PZTs this relationship depends on many structural and other experimental characteristics as stated in the aforementioned references. For this reason, it can be complicated to analytically obtain the relationship voltage/force of a PZT acting as a dynamic exciter of a generic tested structure.

## 3. Experimental Set-Up and Signal Analysis

The use of PZT-patches to perform Experimental Modal Analysis has been tested in a structure consisting of a circular disk hung with a thin rope. The structure has been excited with a PZT-patch glued on its surface and with a calibrated Hammer. Furthermore, the advantages of using PZTs as exciters for submerged structures are tested by making the same experiment for the structure totally immersed in water.

### 3.1. Equipement Used

#### 3.1.1. Tested Structure

The tested structure consists of a stainless steel disk with a diameter of 200 mm. The thickness of the disk is 8 mm and the total mass approximately 7.6 kg.

#### 3.1.2. PZT-Patch

**Figure 1:**
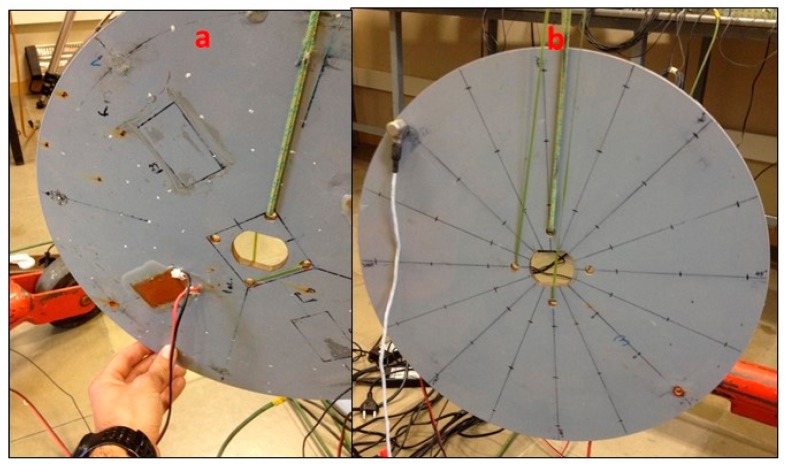
(**a**) Tested structure with installed PZT; and (**b**) installed Accelerometer (back side of the disk).

The Patch (PI-876-A12) has been glued on the disk with an epoxy component (LOCTITE 9466). Since the disk will also be tested submerged in water, the PZT is covered by a transparent epoxy component (LOCTITE 9496). A view of the disk with the installed PZT can be seen in Figure 1a. The black cable ends at the negative terminal of the PZT and the red one at the positive. The mass of the PZT is approximately 3 g.

#### 3.1.3. Accelerometer

To measure the dynamic response of the disk, a submergible Accelerometer Dytran 3006A (Dytran, Chatsworth, CA, USA) (mass 12 g) with a sensitivity of 100 mV/g has been used (Figure 1b).

#### 3.1.4. Instrumented Hammer

To obtain the reference FRF, the calibrated Hammer Dytran 9722A2000 (Dytran, Chatsworth, CA, USA) with a sensitivity of 2 mV/N has been used. The mass of the Hammer is approximately 180 g.

#### 3.1.5. Signal Generator and Amplifier

When using the PZT as exciters, a previous amplification of the signal is needed. The range of the actual Patch is from −100 V to 250 V. Since the signal generator NI-9263 creates an analogic signal from −10 V to 10 V, the amplifier OEM 835 (gain 25) is used.

#### 3.1.6. Acquisition System

**Figure 2:**
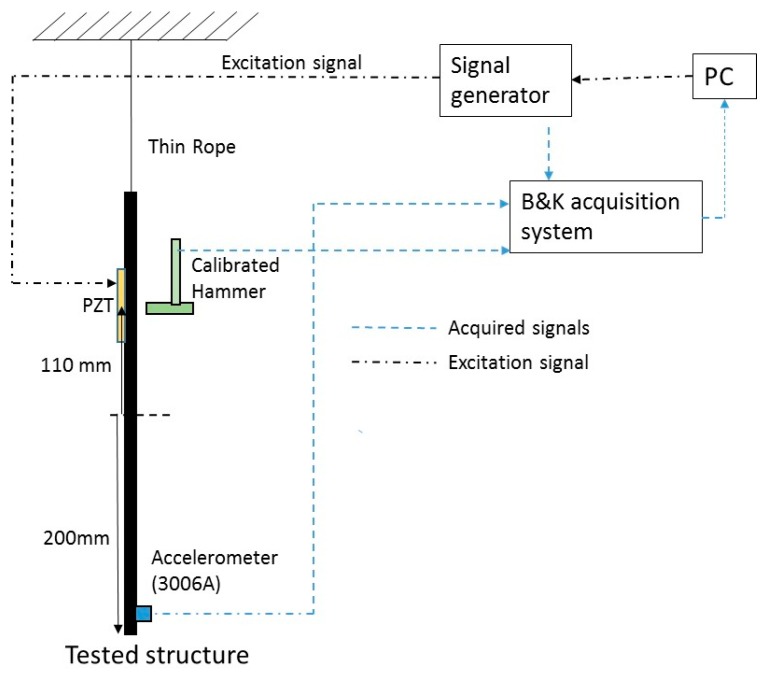
Equipment used.

The signals are simultaneously acquired using a B&K LANXI5023 (Bruel and Kjaer, Nærum, Denmark) module with 12 channels. The signals have been acquired with 4096 samples/s in order to analyze the first modes of the structure (analyzed band 0 Hz–1000 Hz). A scheme of the equipment used can be seen in Figure 2.

### 3.2. Excitation Characteristic and Signal Analysis

#### 3.2.1. Chirp (PZT)

A chirp signal consists of a rapid variation of the frequency of a pure sinusoidal signal. If the frequency bandwidth excited starts at 0 Hz, the signal can be expressed as:

(7) $$V\left(t\right)=A\cdot \sin\left(\beta \cdot t^{2}\right)$$

*V*(*t*) is the signal generated with the signal generator (unit volts). *A* is the amplitude of the signal (for the NI9263 the maximum *A* is 10 V), which is kept constant for all the excited frequencies (Equation (7)). β is a parameter that controls the frequency variation of the signal (higher β means a faster variation in the signal frequency). Adjusting β and selecting the appropriate t, the frequency band excited can be controlled [13].

In this case, a chirp signal of 8 s varying the frequency from 0 to 1000 Hz is used. The disk is excited 5 times in this way in order to obtain an averaged FRF and also to calculate the coherence function [2].

#### 3.2.2. Sweep

In previous studies performed by the same authors, a sweep excitation was used to excite the structure instead of a chirp [13,15,16,17]. This excitation has exactly the same equation as for the chirp but with a substantially lower β. This implies, that the total time to excite the same frequency bandwidth is substantially larger, and that in order to have an estimation of the FRF the peak hold method technique has to be used as explained in those references. The advantage of such a slow chirp is that transients phenomena can be clearly analyzed than with a chirp [16]. The suitability of this excitation characteristic to determine the FRF is also discussed in this paper. The signal used lasts 250 s varying the frequency from 0 to 1000 Hz.

#### 3.2.3. Hammer

The disk is impacted 5 times on the middle of the PZT but on the opposite side (Figure 2). The FRF is averaged with this 5 impacts and the coherence function is also obtained.

#### 3.2.4. Signal Analysis

**Figure 3:**
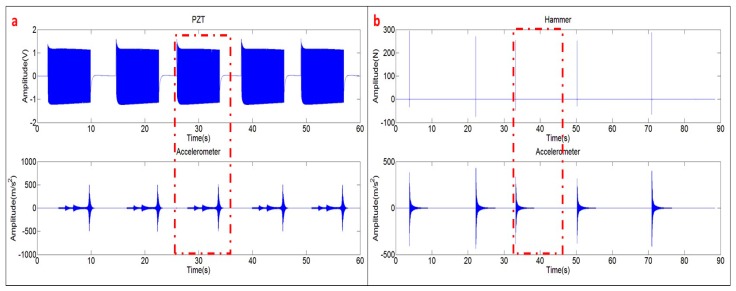
Time signals: (**a**) chirp excitation (PZT) and response; and (**b**) hammer excitation and response.

For all the excitation methods, the signals are windowed with a window of 16 s (therefore resolution in frequency of 1/16 Hz). Each window starts triggered by the excitation signal (Figure 3). When exciting the structure with the PZT, the time signals of the Accelerometer and PZT are weighted with a transient signal of 10 s in order to select just one “chirp” (Figure 3). When exciting the disk with the Hammer, the time signals of the Accelerometer and Patch are weighted with a transient window of 16 s.

For each average, one sample of the vectors $\ddot{x}\left(t\right),\;F\left(t\right)$ (Hammer excitation) and V(t) (PZT excitation) is obtained. These are transformed in the frequency domain by means of the Fast Fourier Transform (FFT) and the FRF H1 calculated averaging according to standard methods [2]:

(8) $${\left[H\left(j\omega \right)\right]}_{1}=\frac{\sum_{1}^{Na\;}\left\{X\left(j\omega \right)\right\}\cdot {\left\{F\left(j\omega \right)\right\}}^{*}}{\sum_{1}^{Na}\left\{F\left(j\omega \right)\right\}\cdot {\left\{F\left(j\omega \right)\right\}}^{*}}$$

where {F(jω)}, {X(jω)} are the discrete vectors obtained after applying the FFT to each sample. Both vectors have a frequency resolution 1/16 Hz (window of 16 s), going from 0 Hz (continuous component) to 1000 Hz. ${\left\{F\left(j\omega \right)\right\}}^{*}$ is just the conjugation of {F(jω)} and *N_a_* the number of averages (5 in this case).

It has to be noticed that calculating the FRF in this way will estimate the true FRF for the Hammer excitation, since the relation will be displacement/force. For the PZT, Equation (8) can be also used (substituting V(jω) for F(jω)) but this will give just a transfer function ((m/s^2^)/V).

**Figure 4:**
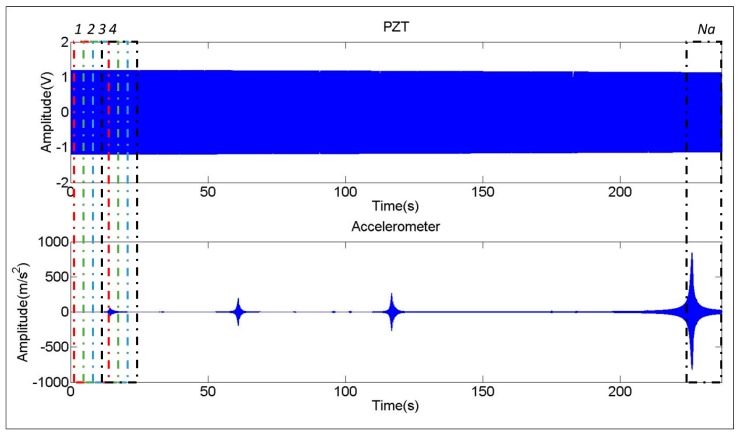
Sweep excitation (PZT): excitation and response.

The analysis of the sweep excitation is performed with the peak hold method, which records the maximal amplitude of each spectral line, when analyzing all the averages. To obtain the averages again, a window of 16 s is used. The window is shifted 0.8 s from average to average (95% overlap). In this way *N_a_* averages are obtained (375 averages with 300 s of signal and 95% overlap of the windows) (see Figure 4). The vectors ${\left\{F\left(j\omega \right)\right\}}_{max},\;{\left\{X\left(j\omega \right)\right\}}_{max}$ are built up with the maximum value obtained for every spectral line in one of the *N_a_* averages considered. Therefore, the FRF of Equation (8) is calculated with these resulting vectors and without averaging.

### 3.3. Tests Performed (Configurations)

#### 3.3.1. Structure Suspended in Air

The disk is excited, according to the aforementioned techniques, hanging surrounded by the atmospheric air (Figure 1).

#### 3.3.2. Structure Submerged in Water (Infinite Medium)

When the structure is submerged in a heavy fluid, the dynamics of the fluid itself greatly influences the dynamic response of the structure as reported in many studies [23,26,27,36,37]. In order to determine the FRF in this situation, the same procedure is repeated. This has also the experimental interest to see if the PZTs have been working properly when they are totally submerged, i.e., that the epoxy shell is working good and that the method proposed in the next section is still valid for this case.

#### 3.3.3. Structure Submerged in Water with a Nearby Rigid Wall

**Figure 5:**
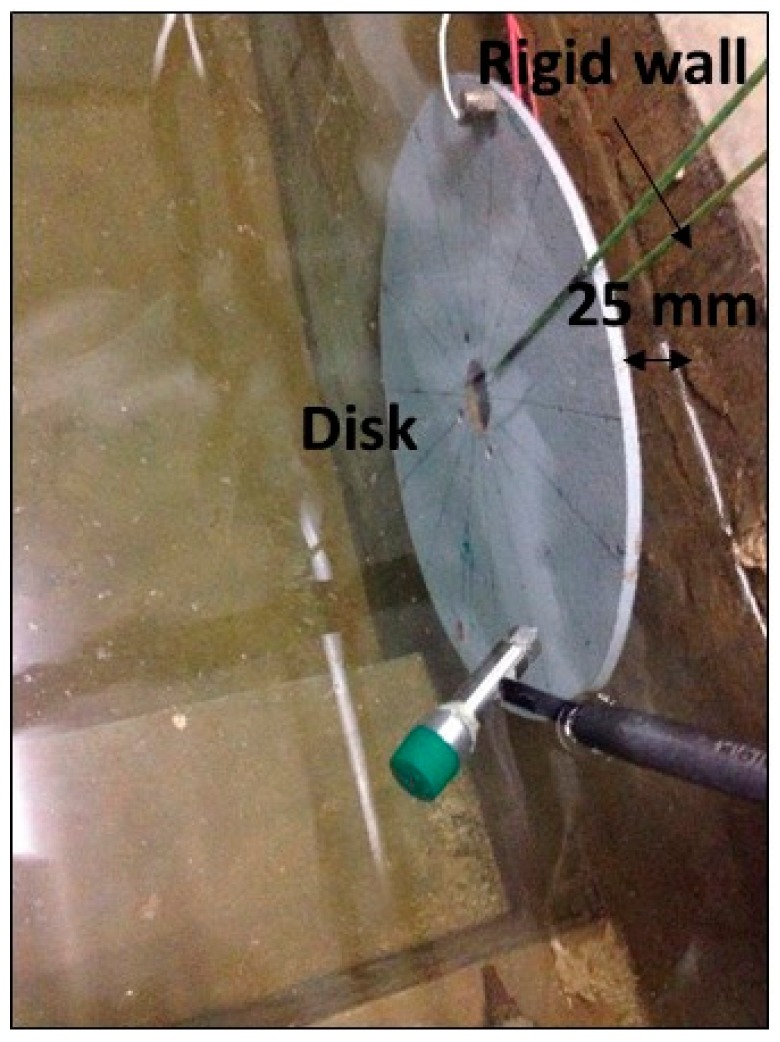
Tested Structure (disk) submerged in water with a nearby rigid wall.

The effect of the surrounding fluid is even more important for the case that the tested structure is close to rigid walls as reported also in many studies [15,28,31,38,39,40]. Therefore, it is desired to see possible changes of the FRF due to the induced motion on the structure due to a Hammer impact in this situation. For this reason, the disk has been excited with the two aforementioned methods, when it is submerged in water and with a nearby rigid wall (Figure 5).

## 4. Methodology to Determine the FRF of a Structure by Means of PZT Excitation

This section aims to show a methodology to obtain the FRF when an arbitrary structure is excited with a PZT. As mentioned in Section 2, plenty of studies have shown that natural frequencies and mode shapes are well determined when using PZTs for many types of structures vibrating in air or in water. Nevertheless, these studies do not discuss how to determine the damping ratios and scaling factors which completely define the FRF.

In order to estimate the modal parameters and the FRF when the disk is excited with the PZT, the transfer function ((m/s^2^)/V) obtained with the PZT is compared with the FRF ((m/s^2^)/N) obtained with the calibrated Hammer (reference FRF).

**Figure 6:**
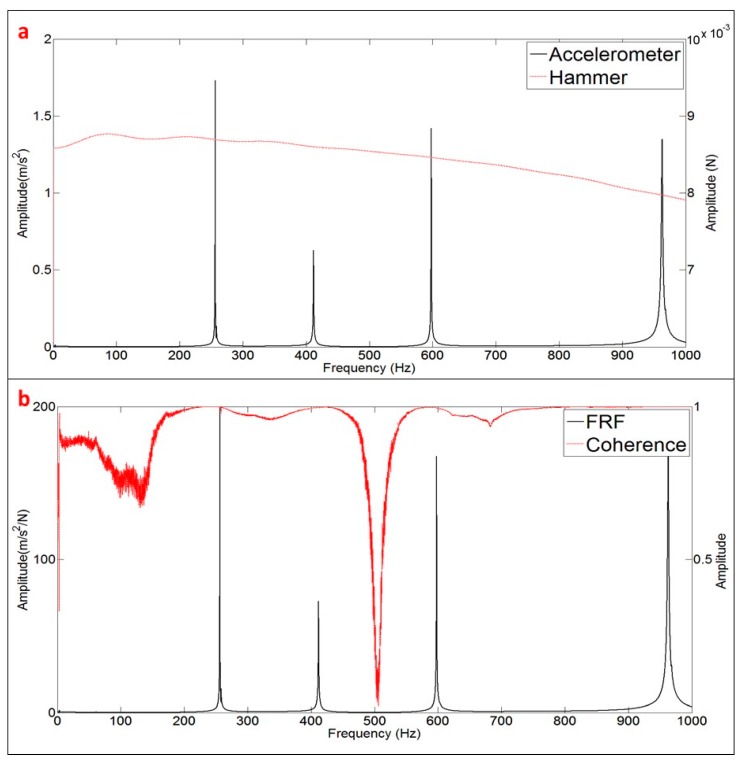
Obtaining the FRF: (**a**) One average of the frequency signals of Accelerometer and Hammer after applying the FFT; and (**b**) FRF estimated with 5 averages (impacts) and coherence.

The FRF is calculated according to Equation (8). Figure 6a shows the signals of the Accelerometer and Hammer in the frequency domain, after applying the FFT. Both signals are analyzed until 1 kHz, including the first four modes of the tested structure (see peaks of the Accelerometer signal ($\left\{\ddot{X}\left(j\omega \right)\right\})$. The response of the Hammer ({F(jω)}) shows that the structure has been properly excited, with the same order of excitation force in all the frequency bandwidth. Figure 6a shows just one average triggered by the Hammer (one impact as seen in Figure 3b).

Figure 6b shows the FRF obtained after averaging the 5 impacts (Figure 3b). The quality of this FRF is evaluated with the coherence function [2]. A value of this function close to the unity (for one frequency) means a cause/effect relationship between excitation and response (repetitive experiments) and as a consequence, good quality of the measurements. Evaluating this indicator close to the resonant peaks (Figure 3b), it can be concluded that the FRF estimated with the Hammer excitation can be used as the reference FRF for further comparison with the function estimated with the PZT-excitation.

The transfer function of the PZT (chirp excitation) is calculated with the same mathematical procedure (Equation (8)) changing the vector of force F(jω) by the vector of electrical signal V(jω). Therefore, this transfer function will not be the truth FRF. Therefore, since the relation within V(jω) and F(jω) is not known for the PZT (or calibration of the PZT in force), the way to proceed will be to compare the modal parameters estimated with this transfer function and with the modal parameters estimated with the real FRF (Figure 6).

**Figure 7:**
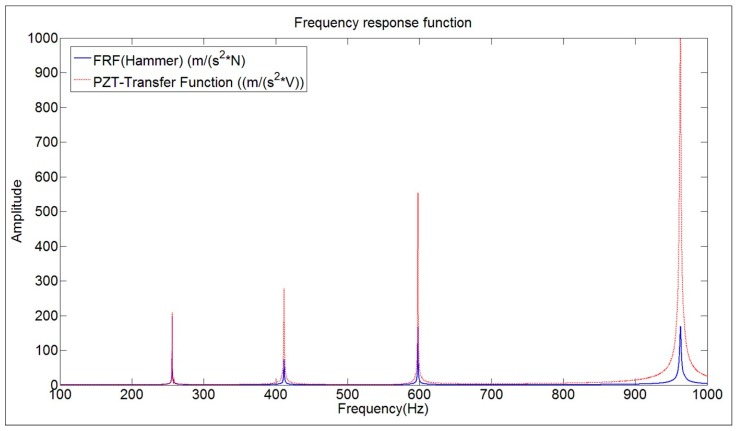
Comparison of the FRF obtained with the Hammer and the transfer function ((m/s^2^)/V) obtained with the PZT (chirp excitation).

Figure 7 shows both functions in the same graph. At first sight, it can be seen that both functions show the same peaks but with an increasing relative amplitude of the PZT with respect to the FRF. This means that the dynamic force of the Patch increases with frequency, since the amplitude of the electrical signal is kept constant for all the frequencies (see chirp in Equation (7)).

### 4.1. Natural Frequencies

Natural frequencies are obtained evaluating the peaks of Figure 7, for the FRF and for the transfer function (PZT- chirp excitation). The values of the natural frequencies estimated with the peaks are shown in Table 1. As in other studies [13,17,28], it is shown that natural frequencies can be properly estimated by using PZTs. Consequently, for the analyzed case, the effect of the Hammer mass (load mass effect [7]) was negligible and obviously also the mass of the PZT (which is much more lighter). Nevertheless, for very light structures, this effect could appear when exciting the structure with a relative heavy transducer, reducing the natural frequencies of the structure (increasing apparent mass) [7]. In such case, the natural frequencies would be more precisely determined by using PZTs.

### 4.2. Mode Shapes

For $\omega =\omega_{r},$ the deformation of the structure is dominated by the mode shape ${\left\{\vartheta \right\}}_{r}$ [1,2]. This modal parameter is a vector of dimension *n* which is differently for each mode *r*. The Mode Shape *r* can be obtained evaluating one column of the matrix *H*(*j*ω) for $\omega =\omega_{r}$, where the contribution of the rest of the modes is almost negligible (or the contribution of the mode *r* is maximum). For $\omega =\omega_{r}$, Equation (3) can be rewritten as:

(9) $$\left[H\left(j\omega_{r}\right)\right]\approx \frac{j2\omega_{r}Q_{r}{\left\{\vartheta \right\}}_{r}{{\left\{\vartheta \right\}}_{r}}^{t}}{\left(\theta_{r}^{2}\right)-2\theta_{r}j\omega_{r}}=K^{\prime }\cdot [\begin{array}{ccc} \vartheta_{1,r}\cdot \vartheta_{1,r} & \begin{array}{cc} \vartheta_{2,r}\cdot \vartheta_{1,r} & \ldots \end{array} & \vartheta_{n,r}\cdot \vartheta_{1,r}\\ \vartheta_{1,r}\cdot \vartheta_{2,r} & \begin{array}{cc} \vartheta_{2,r}\cdot \vartheta_{2,r} & \ldots \end{array} & \vartheta_{n,r}\cdot \vartheta_{2,r}\\ \begin{array}{c} \vdots \\ \vartheta_{1,r}\cdot \vartheta_{n,r} \end{array} & \begin{array}{c} \begin{array}{cc} \vartheta_{2,r}\cdot \vartheta_{3,r} & \ldots \end{array}\\ \begin{array}{cc} \vartheta_{2,r}\cdot \vartheta_{4,r} & \ldots \end{array} \end{array} & \begin{array}{c} \vdots \\ \vartheta_{n,r}\cdot \vartheta_{n,r} \end{array} \end{array}]$$

where $K^{\prime }$ is a constant factor (in the complex plane) that equally affects all the elements of the matrix $H\left(j\omega_{r}\right)$. Therefore, one column of $H\left(j\omega_{r}\right)$ is an estimation of the Mode Shape *r* ${\left\{\vartheta \right\}}_{r}=\left\{\begin{array}{cc} \vartheta_{1,r} & \begin{array}{cc} \ldots & \vartheta_{n,r} \end{array} \end{array}\right\}$ (scaled by a different factor depending on the column selected). According to the definition of H(jω), a column of H(jω) means to have the response of the structure for the *n* different points when the excitation is fixed at one point (rowing accelerometer method). According to Maxwell reciprocity principle [1,2], this is also equivalent to have one fixed point for the response and *n* different points for the excitation (rowing hammer method).

In this case, since the position of the excitation is fixed (PZT is glued to the disk), the rowing accelerometer method has been used, measuring the response in 16 equidistant points on the periphery of the disk, since the several first mode shapes have the most relevant deformation in this part [13,15,28]. For each measurement position, the disk has been excited with the instrumented Hammer and with the PZT.

**Figure 8:**
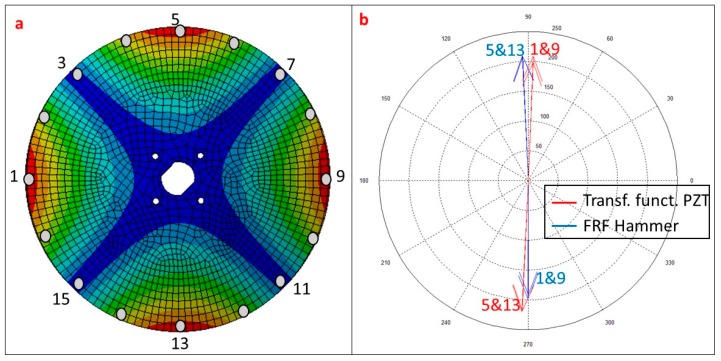
(**a**) First Mode Shape of the analyzed structure with measured points; (**b**) Representation of $H\left(j\omega_{r}\right)$ for the points with maximal deformation (Hammer and PZT).

Each element of the column of $H\left(j\omega_{r}\right)$ (H(jω) evaluated in $\omega_{r}$) is just a complex number that can be represented in amplitude and phase. Figure 8a shows the first Mode Shape of the structure with the experimentally measured points. $H\left(j\omega_{r}\right)$ is represented for the points with maximal deformation (1, 5, 9, 13) of the current mode (Figure 8b). These points are shifted approximately 180° when comparing the PZT function and the FRF obtained with the Hammer. This phase shift occurs because the Patch is acting on the opposite side than the Hammer (see Figure 2).

To compare the columns $H\left(j\omega_{r}\right)$ obtained with the Hammer and with the PZT (vectors of complex numbers), the MAC indicator is used [1,2]. A MAC-value close to 100% means two parallel vectors in the complex plane (differentiated only by a complex constant factor). The values close to 100% (Table 1) show that, again, when using the transfer function estimated with the PZT, the mode shapes can be also obtained.

### 4.3. Damping Ratio

In the literature, it has been proved that natural frequencies and mode shapes can be properly estimated when using PZTs. Nevertheless the determination of damping ratios has not been deeply discussed yet. For this purpose, in this paper, the damping ratios obtained with the PZT-Transfer Function and with the true FRF obtained with the Hammer are compared. Damping Ratio ($\varepsilon_{r}=\theta_{r}/\omega_{r})$ can be estimated with the half power method [1,2]. For each mode *r* (or peak of the FRF), the following calculation has to be performed:

(10) $$\varepsilon_{r}=\frac{\omega_{b,r}-\omega_{a,r}}{2\omega_{r}}$$

**Figure 9:**
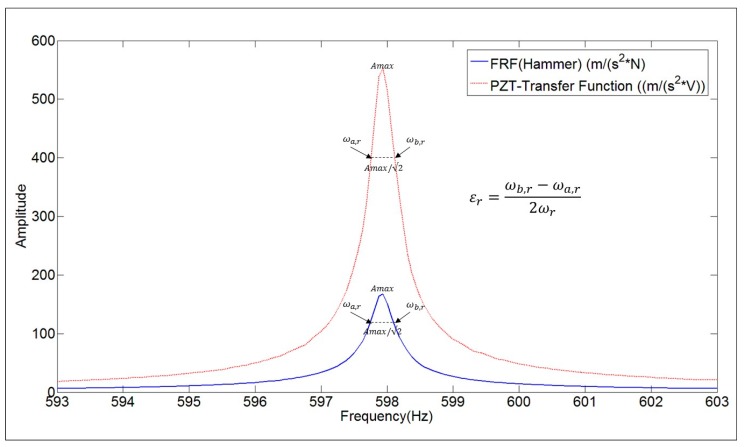
Damping Ratio estimation with the half power method for the FRF and for the transfer function ((m/s^2^)/V) obtained with the PZT (chirp excitation).

The values $\omega_{b,r},\omega_{a,r}$ determine the frequency band around $\omega_{r}$ (Natural Frequency), where the amplitude of the FRF is $A_{max}/\surd 2$ (see Figure 9). Although, for this mode (3rd mode of Table 1), the amplitude of both functions is completely different, the Damping Ratio is approximately the same.

The numerical value of the damping ratios estimated with both functions for all the analyzed modes, are shown in Table 1. From the results, it can be concluded that the damping ratios can be properly estimated when using the transfer function obtained with the chirp excitation made with the PZT ((m/s^2^)/V) (numerical discrepancy of 5%).

In fact, it can be easily demonstrated that damping ratios could be also estimated with the half power method using directly the response in the frequency domain ({X(jω)}, $\left\{\dot{X}\left(j\omega \right)\right\}\;or\;\left\{\ddot{X}\left(j\omega \right)\right\}$) instead of using the FRF, if the force in the narrow frequency band $\left(\omega_{a,r}:\omega_{b,r}\right)$ can be assumed as constant. For instance, for an impact excitation this assumption is generally valid. Therefore, the results obtained with the transfer function of the PZT demonstrates that, although the force of the patches varies with the frequency (as commented before from the deductions of Figure 7), it can be assumed that this force amplitude is approximately constant in the narrow frequency bandwidths that define the damping ratios.

**Figure 10:**
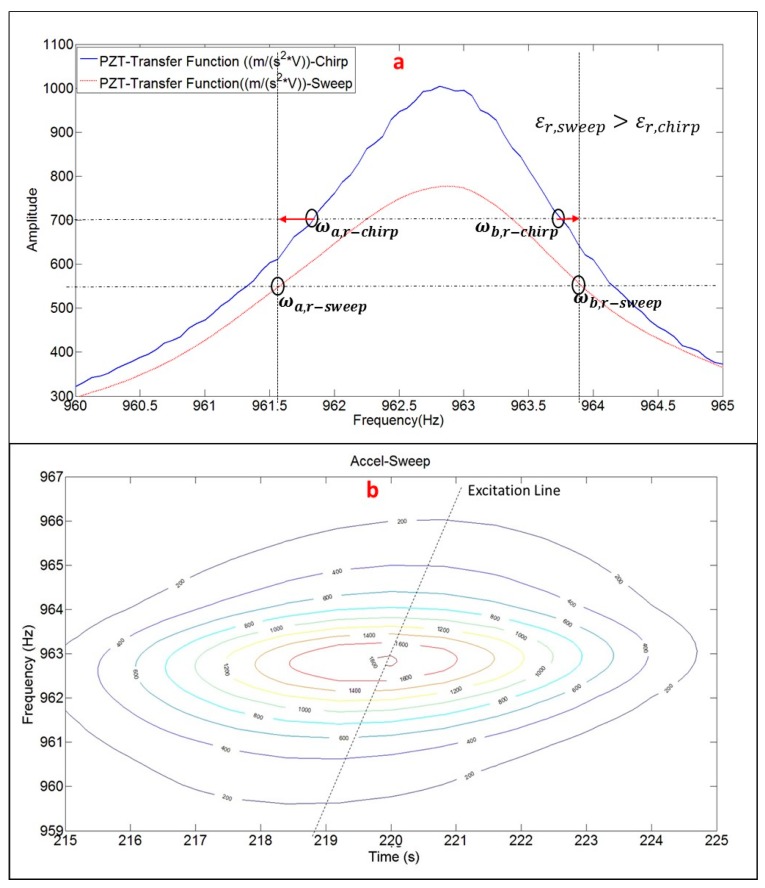
(**a**) Comparison PZT-Transfer function within Sweep and Chirp; and (**b**) detailed Time–Frequency representation of the peak hold method analysis for the sweep excitation.

Until now, all the comparisons and conclusions have been made for the chirp excitation. Now, once the chirp excitation has been validated to obtain natural frequencies, mode shapes and damping ratios, the Sweep excitation (see Section 3.2.3) is evaluated. For the same vibration mode (Mode 4 in Table 1), the chirp excitation is compared with the sweep excitation (Figure 10a).

As seen in this figure the Damping Ratio estimated with the sweep excitation and peak hold method is larger than with the chirp excitation (since the band $\omega_{a,r}:\omega_{b,r}$ is larger for the sweep function Figure 10a). The red arrows in Figure 10a indicates this overestimation in the frequency bandwidth that define the damping ratio. The reason of this overestimation can be found in Figure 10b where a detailed Time–Frequency representation of the accelerometer signal, when the structure is being excited by the sweep is shown zoomed on the resonance occurrence.

In order to accurately estimate the damping ratio, a good frequency resolution is needed (because the estimation depends on the frequency bandwidth), but this implies a large time window and therefore a poor time resolution [1]. Although the Sweep was a relative slow Sweep (250 s for the bandwith 0–1 KHz), Figure 10b shows that this was not slow enough, since an ideal Sweep (excitation of 16 s for every discrete frequency) would produce the level curves oriented in the direction of the excitation line Figure 10b. As seen in this figure, this is not the case and therefore, the bandwith defining the damping is overestimated because not only a single frequency is excited in such a large time window.

The comparison of the natural frequency, mode shape and damping ratio within these two excitation methods for the third mode is shown in Table 2. As seen in this table, the natural frequency and mode shape are properly obtained when using the Sweep excitation method but the Damping Ratio is overestimated. This trend can be generally extended for the rest of the modes analyzed.

Therefore, although in previous studies it has been demonstrated that the Sweep excitation is a better excitation to analyze fast transient phenomena [16], in order to obtain precisely the FRF, the Chirp excitation is a better choice and will be the one used in the rest of the paper.

### 4.4. Proccedure to Estimate the Complete FRF

In the previous sections, it has been demonstrated that most of the modal parameters that define the FRF can be estimated without a previous calibration of the PZT (Table 1). In order to determine completely the FRF, the only remaining parameters are the scaling factors $Q_{r}$ (Equation (3)) that define the amplitude of the FRF.

For this purpose, the procedure proposed here is to use the information obtained experimentally with the PZTs (natural frequencies, damping ratio and mode shapes) and introduce this information in a computational Finite Element Model of the tested structure. These models usually consider the structure as an assembly of masses and spring but without considering the damping [41], since this parameter cannot be directly obtained from the structural properties.

**Figure 11:**
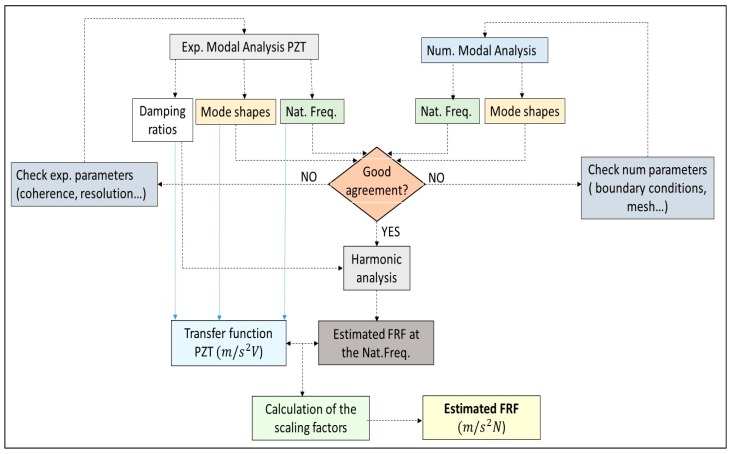
Flowchart of the proposed method to estimate the FRF using PZTs and a numerical simulation model.

Figure 11 shows the flow chart of the methodology proposed. Firstly, Experimental Modal Analysis with the PZT is performed (as explained in the previous sections), obtaining the natural frequencies, mode shapes and damping ratios of the tested structure. At the same time, performing Numerical Modal Analysis on the modeled structure, natural frequencies and mode shapes can be also determined. If there is a good agreement within both numerical and experimental methods, then the numerical model is validated and a numerical harmonic analysis can be performed. Damping ratios have to be estimated experimentally, since they depend on many boundary and environmental conditions [26], that cannot be directly reproduced on the numerical model. Therefore, the damping ratios obtained experimentally (with the PZT chirp excitation) are introduced in the numerical harmonic analysis set-up [41]. Exciting the structure at the frequency bandwidths around the modes of interest, the real amplitude of the FRF around these modes can be estimated. In this way, comparing these amplitudes with the amplitudes of the transfer function obtained with the PZT, the scaling factors (one factor for each mode, see Equation (3)) are obtained and the full FRF estimated.

For the present test, the structure is modeled and analyzed with ANSYS v.16 (ANSYS, Canonsburg, PA, USA), which permits to introduce the experimental damping ratios in the harmonic response test. Firstly, with Numerical Modal Analysis (without damping) it has been checked, that the natural frequencies obtained experimentally and numerically have a difference of less than 2.5% and that the MAC within the mode shapes obtained with numerical and experimental tests (PZT) is higher than 98% for all of the analyzed modes (see Table 3). Therefore, the computational model of the structure is validated.

**Figure 12:**
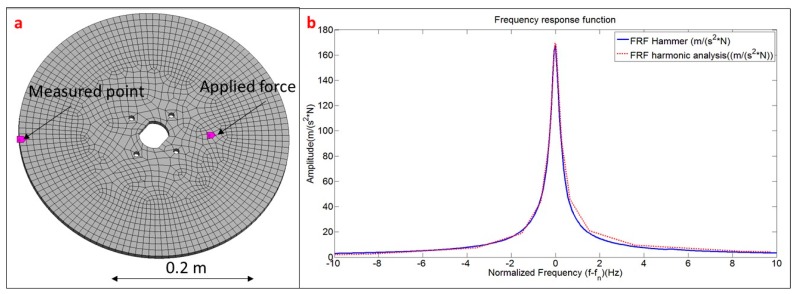
(**a**) Simulation model including the position of the force and the measurement point; and (**b**) application of the harmonic response compared to experimental result for the 3rd mode.

Once the numerical model is validated, the damping ratios obtained with the PZT transfer function are introduced in the numerical harmonic analysis set-up. To validate this method and to properly estimate the scaling factors of the PZT, the force is applied on the middle of the PZT surface and the measuring point on the position of the accelerometer (Figure 12a). For each simulation step, the structure is excited with a pure sinusoidal force, so many cycles as necessary to guarantee a steady harmonic solution. For each new simulation step the amplitude of the force is kept constant and the frequency increased. In this way, analyzing in detail the frequency bandwidths around the different resonances, the amplitude of the FRF for these resonances has been estimated. Figure 12b shows the comparison between the FRF estimated through the procedure explained in this section and the reference FRF obtained with the Hammer. As seen in this figure, the experimental FRF and its estimation with the proposed methodology show very good agreement.

This methodology is applied to estimate the amplitude of the FRF at the natural frequencies of all the analyzed modes below 1 kHz. The scaling factors to correct the transfer function obtained with the PZTs (Figure 7) are obtained comparing the amplitudes of that function with the amplitudes of the estimated FRF for each mode. This means the transfer function ( PZT-Transfer Function in Figure 7) is reescaled to have the same amplitude at the natural frequencies than the estimated FRF obtained with the proposed method. Therefore, for each mode there is a scaling factor that corrects the FRF in the vicinity of the corresponding mode according to Equation (3).

**Figure 13:**
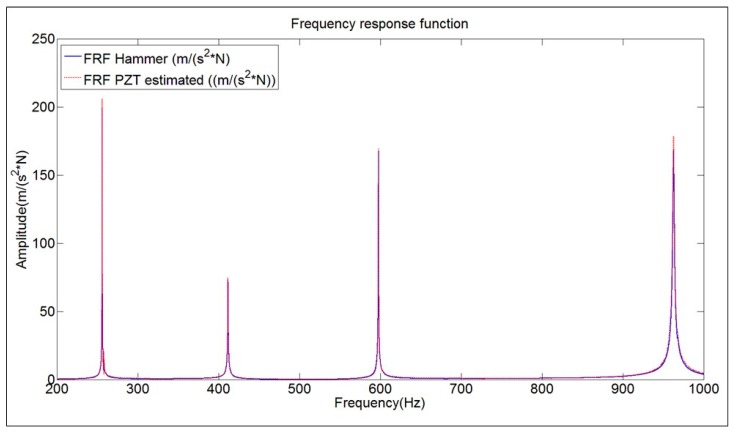
Comparison FRF obtained with Hammer and FRF estimated with the PZT combined with the proposed method. Structure suspended in air.

Figure 13 shows the reescaled transfer function of Figure 7 (FRF PZT estimated), according to the calculated scaling factors, compared with the reference FRF obtained with the Hammer. Now, both functions have approximately the same amplitude for all the frequency range below 1 kHz (discrepancy of less than 6% of amplitude at the natural frequencies).

Note that the reference FRF of the Hammer has been used here just to validate the methodology. Nevertheless, once this methodology has been validated, it is not necessary to have this function. Therefore, as a conclusion, the FRF could have been estimated with this procedure without using a calibrated transducer, such as the Hammer. Furthermore, a previous calibration of the PZT was also not necessary.

## 5. Determination of the FRF for Submerged Structures with Nearby Rigid Walls

In the previous section, the FRF of the sturcture vibrating in air has been succesfully obtained using the PZT and applying the proposed methodology in this paper. Nevertheless, the interest of this method for accesible structures vibrating in air is likely limited, since clasical exciters (Hammer) are easier to use. However, in this section, it will be shown that in order to accurately determine the FRF of a submerged and confined structure, PZTs are much better exciters if the proposed methodology is followed.

### 5.1. FRF of the Structure with Infinite Water Medium

First, in order to validate the correct operation of the PZTs in water medium, i.e., that the epoxy covering on the PZT is properly isolating the PZT from the water, the tested structure has been totally submerged in water and with large distances disk to rigid walls (usually in the literature this situation is considered as infinite medium). In this situation, a small increase/decrease of the gap to the rigid walls or to the free surface does not change the added mass effect and therefore the natural frequencies and damping ratios remain constant [15,28,31].

The natural frequencies and damping ratios estimated with the hammer excitation and with the PZTs are compared in Table 4. Note that the natural frequencies have been drastically reduced due to the added mass effect of the fluid [23,31,38].

In this case, the natural frequencies and damping ratios are still aproximately the same when comparing both methods. As a consequence, the induced motion of the disk due to the impact of the Hammer does not affect the dynamic response of the structure in this situation (impacts of aproximately 200 N).

**Figure 14:**
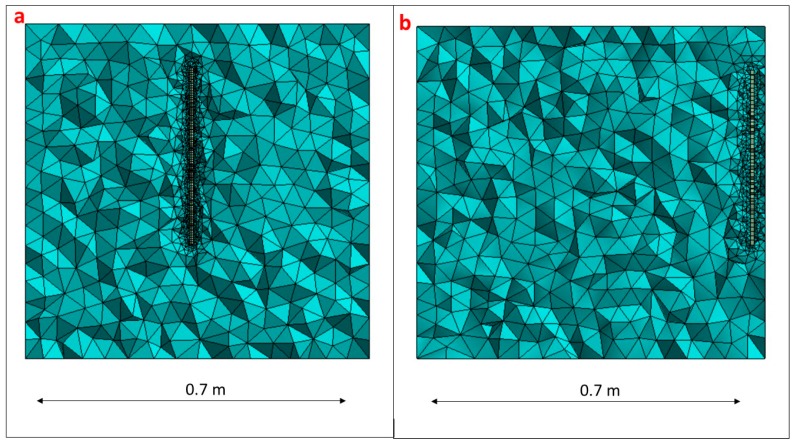
Simulation model used: (**a**) Disk with “infinite” water medium; and (**b**) disk close to rigid wall (25 mm).

To obtain the FRF in this case, the simulation model has been modified. Now, not only the disk, but also the surrounding medium has to be considered. The water has been modelized with acoustic elements and the whole system has aproximately 100,000 elements. A mesh sensitivity analysis was performed in order to assure convergence in the natural frequency values. With the mesh tested the discrepancy between the natural frequencies obtained with the PZT or Hammer an the natural frequencies obtained with the simulation model is less than 3% for the considerd modes and therefore a harmonic analysis with the damping ratios obtained experimentally can be performed to estimate the FRF (see proccedure in Figure 11 and previous section). A detailed view of the simulation model used for this case can be seen in Figure 14a.

**Figure 15:**
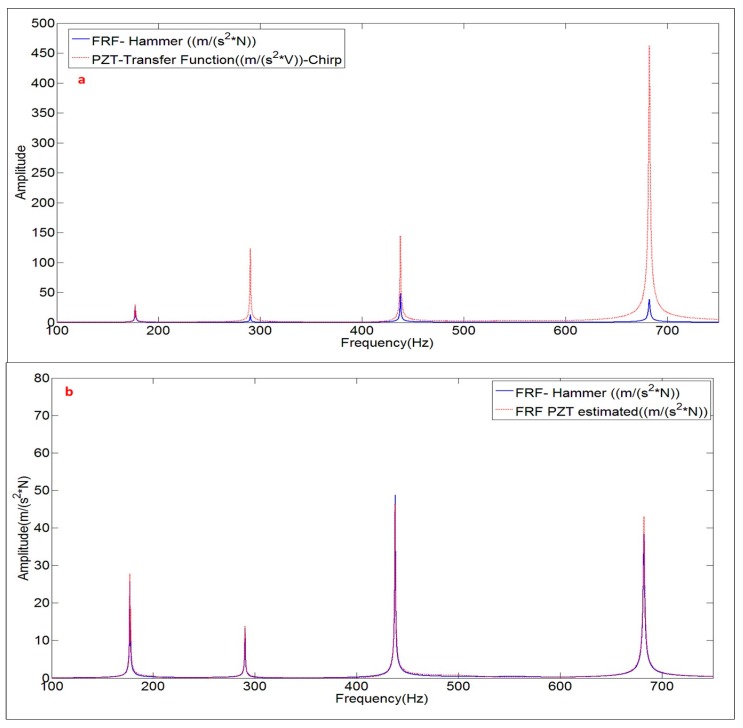
(**a**) Comparison PZT transfer function with FRF; and (**b**) comparison FRF estimated with the PZT and FRF obtained with the Hammer. Structure with infinite water medium.

The comparison between the FRF obtained with the Hammer and the Frequency Function obtained with the PZT, for the disk submerged in “infinite water” can be seen in Figure 15a. The analysis of the amplitudes, leads to the same conclusion than when the structure was hung in air, i.e., the dynamic force of the PZT increases when increasing the frequency, since the voltage is kept constant.

Using the proccedure explained in Figure 11, the FRF estimated with the PZT excitation is compared with the FRF obtained with the Hammer (Figure 15b). Again, the FRF is well aproximated with the proposed method when using PZTs and therefore the correct work of the PZTs in water is experimentally proven.

### 5.2. FRF of the Structure Close to a Rigid Wall

This case, which is representative of some engineering structures such as hydraulic turbines, becomes the most challenging one since the motion induced with the impact that is made to obtain the FRF, could affect the experimental estimations, since the dynamic response of the structure is very sensitive to the distance structure–rigid wall and its derivatives (see Section 2.2). Particularly, as reported in some studies, the damping ratios of such structures are greatly affected by the gap structure–rigid wall [28,32]. In this case, the use of a classical exciters such as a Hammer may modify the dynamics of the fluid itself and consequently the hydrodynamic Damping [26]. Therefore, the use of light, thin and small exciters, such as PZTs, becomes of relevant interest to accurately determine the FRF in these cases.

**Figure 16:**
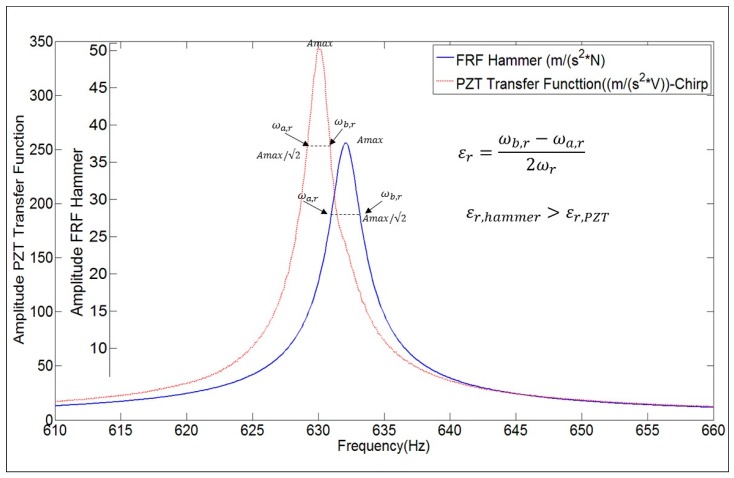
Natural Frequency and Damping estimation of the fourth mode of the disk submerged in water close to a rigid wall.

The experimental tests made with the disk submerged and with 25 mm to a rigid wall (Figure 5) confirm this fact. Figure 16 shows the FRF estimated with the Hammer and the Frequency Function obtained with the PZT for the fourth mode.

From this figure, comparing the Hammer excitation method and the PZT excitation, it can be seen that the natural frequency calculated is approximately the same for both excitation methods (less of 1% of difference) but the damping ratio is much higher for the Hammer case.

According to the explanation at the begin of this section and according to the hydrodynamic damping theory [26,42], the phenomena could be that when impacting the structure with the Hammer, one part of the structure slightly moves in the water towards the rigid wall. As mentioned in Section 2 and also proven experimentally in many studies [28,29,32,38], when the structure is very close to a rigid wall, a small change in this gap or its derivatives will affect the natural frequencies and the damping ratios. According to the experimental results, in this case, the change in the gap structure–rigid wall due to the impact is almost negligible, since the natural frequencies are approximately the same. Nevertheless, the high increase in the Fluid Damping when using the Hammer, suggest that the induced velocity due to the impact has a great effect on the damping ratio.

The same trend in the natural frequencies and damping ratios is obtained for the rest of the modes (Table 5), i.e., natural frequencies are approximately the same and damping ratios are much higher when the structure was excited with the Hammer. Therefore, as a general conclusion here, it can be stated that the use of the Hammer has slightly modified the boundary conditions affecting the estimation of the modal parameters and specially the damping ratios, which are very sensitive when the structure is close to a rigid wall [28]. This induced motion on the structure due to the impact is of course undesired, since it means that the use of the exciter is modifying the expected results. For this reason and for the reason that in such situation it is difficult to hit the structure properly with an instrumented Hammer (inaccessibility of the structure), it is preferable to use the PZT excitation for an accurate estimation of the damping ratio.

**Figure 17:**
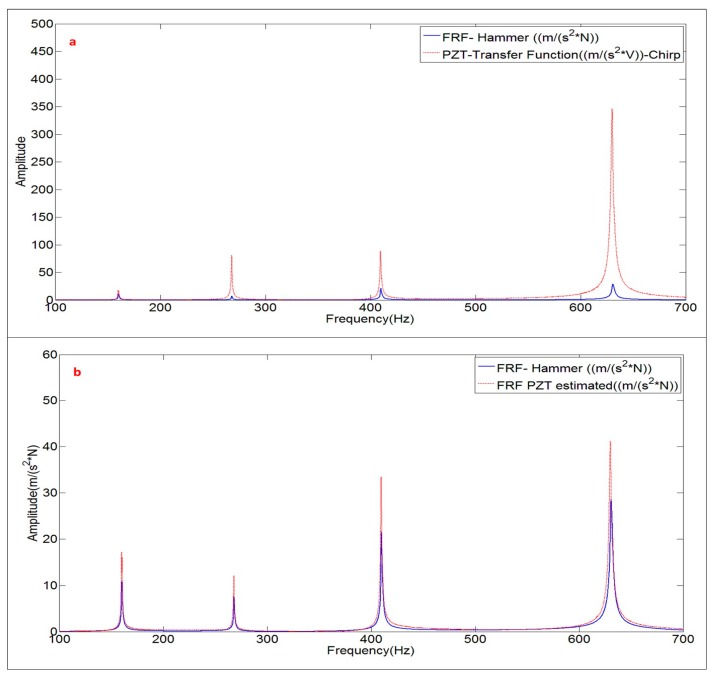
(**a**) Comparison PZT transfer function with FRF; and (**b**) comparison FRF estimated with the PZT and FRF obtained with the Hammer. The Structure is close to a rigid wall.

Finally, the FRF estimated with the PZT is compared with the FRF obtained with the Hammer excitation applying the methodology explained in the previous sections. The simulation model used in the previous section (infinite water) is now modified to have a distance structure–wall of d = 25 mm (Figure 14b). The damping ratios experimentally estimated with the PZT are introduced in the simulation model and the scaling factors calculated. The comparison of the uncorrected function and the FRF estimated with the proposed method against the FRF obtained with the Hammer is shown in Figure 17.

With respect to the air case and infinite water case, where the FRF obtained with the Hammer and the FRF estimated with the proposed method (using the PZT) were approximately the same (Figure 13 and Figure 15), in this case (Figure 17), there are high discrepancies in the amplitudes since damping ratios, which have a great influence on the amplitude of the resonances, are very different when they are determined with the Hammer excitation or with the PZT excitation (Table 5).

This is an important result for this study because it shows that for an accurate estimation of the FRF in such situation the use of PZTs combined with the proposed method will give better results, since the experimentation made with the Hammer clearly modify the boundary conditions of the test.

**Figure 18:**
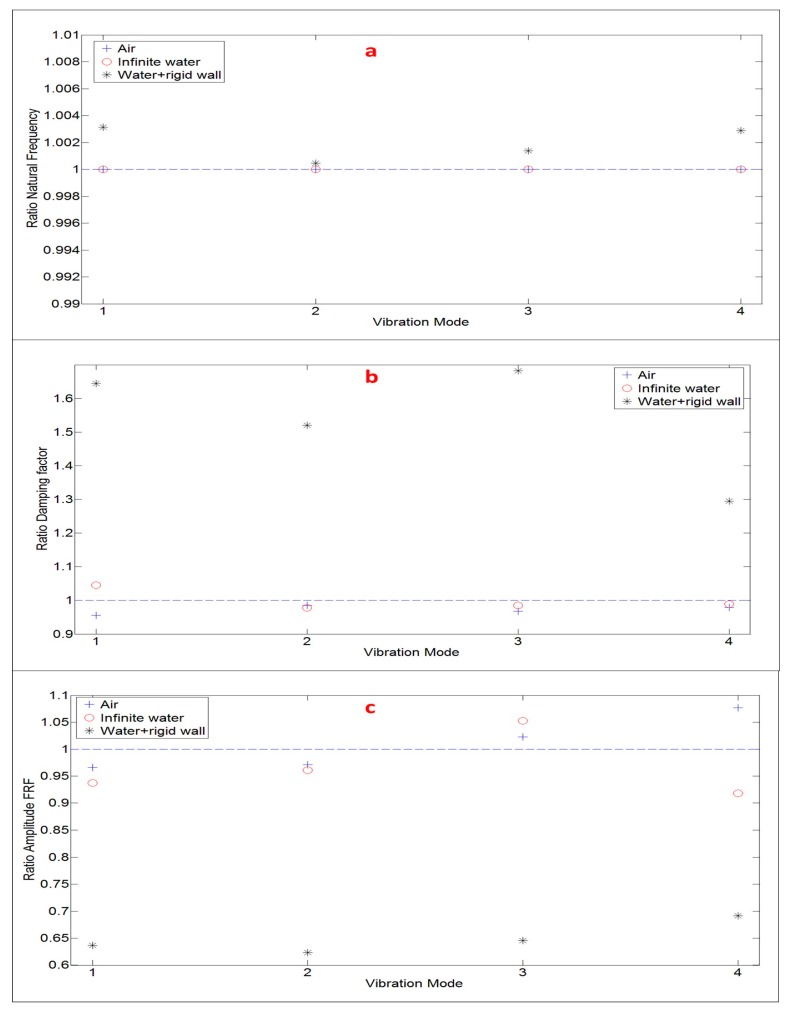
Comparison modal parameters for the four analyzed modes. Ratio modal parameter estimated with the Hammer against modal parameter estimated with the proposed method (using PZTs: (**a**) Natural Frequency; (**b**) Damping Factor; and (**c**) Amplitude FRF.

Finally, in order to summarize the comparisons between estimated FRF (with the proposed method by using PZTs) and FRF obtained with the Hammer (Figure 13, Figure 15 and Figure 17), the modal parameters that define the FRF (natural frequencies, damping ratios and amplitudes) are compared (Figure 18). The ratio modal parameter Hammer/modal parameter estimatedPZT is shown for all the analyzed modes. For all the analyzed modes, the natural frequencies estimated with both methods are practically the same for all the tested situations. For the damping ratios and amplitudes, for the Air and Infinite Water case the difference is less than ±5% and ±8%, respectively. Nevertheless, for the nearby rigid wall case, as commented and justified in this Section, there is an overestimation of the damping ratios for the Hammer method and as a consequence an underestimation in the amplitudes of the FRF function.

### 5.3. Potential Application in a Real Submerged and Confined Structure

**Figure 19:**
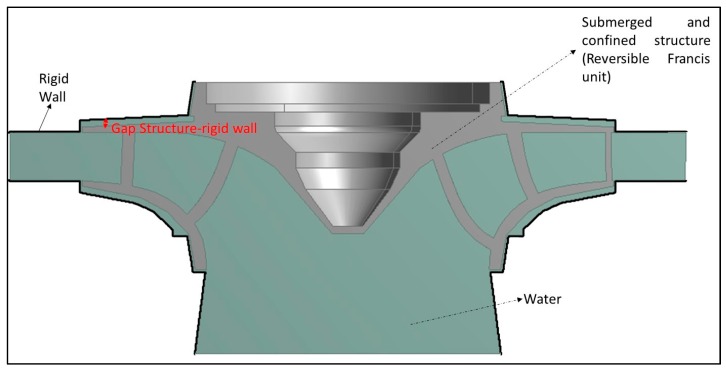
Example of a submerged-confined structure (reversible Francis turbine).

One example of the potential interest of this application is the case of some types of hydraulic turbines, such as Francis units, which are totally submerged and confined with very short distances to external walls (Figure 19). For such structures that are subjected to dynamic loads it is crucial to accurately determine the real FRF and therefore to obtain a realistic structural model with its real operating boundary conditions. This could avoid catastrophic failures and fatigue damages that have been reported for such structures [5,6]. As discussed in the previous section, the use of a classical excitation method such as an impact Hammer may lead to an underestimation in the amplitude of the resonance and to an overestimation of the damping ratios. Therefore, this can have a negative effect for fatigue calculations, if the structure is being excited close to one of its natural frequencies during its operating conditions, since the useful life will be overestimated.

In the past, natural frequencies and mode shapes of these kind of structures have been obtained [17,39,43] but without fully determining the FRF (therefore no damping ratios were obtained). For such structures, PZTs have been proved to be more suitable exciters for physical reasons (inaccessibility of the structure) [17] and this paper shows also that the use of them gives generally more realistic estimations of the damping ratios and therefore more accurately predictions of the amplitude of the FRF than other classical exciters used in EMA.

## 6. Conclusions and Future Perspectives

In this study, a methodology to fully obtain the Frequency Response Function (FRF), or vibration–force relationship, of a generic structure by means of a PZT exciter is developed. The accurate determination of the FRF is of relevant interest in many engineering structures to avoid resonance or fatigue problems that can reduce the useful life of such structures. One particular case is submerged and confined structures, such as hydraulic turbines, which are subjected to high dynamic loads in their real operating conditions. For such structures, the experimental determination of the FRF is a challenging task due to the inaccessibility of the structure and due to the fact that the experimentation done for this purpose (excitation of the structure with an impact) can affect the results. Therefore, for these applications, the FRF could be more accurately determined with exciters such as PZTs which are extremely light, small and thin compared to the tested structure. Nevertheless, with respect to other classical exciters such as instrumented hammers where the relationship between voltage and force is linear and independent from the tested structure, for PZTs this relationship depends on many parameters such as material and thickness of the structure, location of the PZT in the structure or working frequency. Therefore, for a generic case it can be complicated to analytically obtain this relationship.

For this reason, this paper proposes a method that consists of determine the FRF of a general structure by using a PZT and introducing the modal parameters experimentally determined in a validated numerical simulation model of the structure. In this way, the FRF can be obtained by using a PZT and without a calibration of it as a force transducer. The FRF estimated with the proposed method using a PZT and its most characteristic parameters (natural frequencies, mode shapes, and damping ratios) have been compared with the FRF obtained with a calibrated force transducer. Different excitation methods have been discussed and the methodology developed here has been validated.

Particularly, it has been experimentally proven that this method gives good estimations of the FRF for the tested structure vibrating in air and with infinite water medium. Furthermore, it has been experimentally shown that for submerged structures close to rigid walls, the determination of the FRF by means of the explained procedure and using a PZT is more appropriate than the FRF obtained with typical calibrated force transducers used for the same purpose. The reason is not only due to the inaccessibility of the structure as mentioned in previous studies, but also because the use of classical exciters can modify the boundary conditions of the tested structure, modifying also its FRF, as experimentally demonstrated in this paper.

With respect to previous studies, where PZTs have been mainly used as exciters in active/passive vibration of thin structures vibrating in air and in water, where natural frequencies and mode shapes were successfully determined, this paper has also focused on the damping ratios and real amplitudes of the FRF. This has a limited interest for some structures, which are perfectly accessible and where calibrated exciters are easily to be used for the same purpose, but its application can be very important to accurately determine the FRF of real submerged and confined structures such as hydraulic turbines.

## Figures and Tables

**Table 1:** Comparison of the modal parameters (natural frequencies, mode shapes and damping ratios) obtained with the Hammer and with the PZT (Chirp excitation).

Mode	Natural Frequency (Hz)		Mode Shape (MAC %)	Damping Ratio (%)	
	Hammer	PZT		Hammer	PZT
Mode 1	256.19	256.19	99.4	0.032	0.034
Mode 2	411.75	411.75	98.7	0.064	0.065
Mode 3	597.938	597.938	98.9	0.030	0.031
Mode 4	963	962.813	99.3	0.095	0.097

**Table 2:** Comparison modal parameters obtained with sweep and chirp excitation method (3rd Mode).

Excitation	Natural Frequency (Hz)	Mode Shape (MAC %)	Damping Ratio (%)
Sweep	962.813	99.1	0.121
Chirp	962.813	99.3	0.097

**Table 3:** Validation of the numerical model. Comparison of the natural frequencies and mode shapes. Numerical Modal Analysis vs. Experimental Modal Analysis with PZT.

Mode	Natural Frequency (Hz)		Mode Shape
	Simulation	PZT	MAC % (Simulation vs. Experimental PZT)
Mode 1	255.5000	256.19	99.8
Mode 2	421.9600	411.75	98.6
Mode 3	600.2500	597.938	99.1
Mode 4	981.0000	962.813	99.2

**Table 4:** Comparison of the modal parameters (natural frequency and damping ratio) between PZT excitation and Hammer excitation for the structure submerged in “infinite water”.

Modes	Natural Frequency (Hz)		Damping Ratio (%)	
	Hammer	PZT	Hammer	PZT
Mode 1	177.25	177.25	0.115	0.113
Mode 2	290.25	290.25	0.087	0.089
Mode 3	437.75	437.75	0.068	0.069
Mode 4	682.125	682.125	0.108	0.109

**Table 5:** Comparison of the modal parameters (Natural Frequency and Damping Ratio) between PZT excitation and Hammer.

Mode	Natural Frequency (Hz)		Damping Ratio (%)	
	Hammer	PZT	Hammer	PZT
Mode 1	160.563	160.063	0.296	0.180
Mode 2	268.188	268.063	0.19	0.125
Mode 3	410.250	409.688	0.18	0.107
Mode 4	632.063	630.25	0.189	0.146
